# Heterogeneous expression of EPCAM in human circulating tumour cells from patient-derived xenografts

**DOI:** 10.1186/s40364-018-0145-8

**Published:** 2018-10-30

**Authors:** Chiara Agnoletto, Linda Minotti, Laura Brulle-Soumare, Lorenzo Pasquali, Marco Galasso, Fabio Corrà, Federica Baldassari, Jean-Gabriel Judde, Stefano Cairo, Stefano Volinia

**Affiliations:** 1 Department of Morphology, Surgery and Experimental Medicine, LTTA Centre, University of Ferrara, 44121 Ferrara, Italy; 2 Xentech, Paris, France; 3 Dermatology and Venereology Unit, Department of Medicine, Center for Molecular Medicine, Karolinska University Hospital, SE-17176 Stockholm, Sweden

**Keywords:** CTCs, RT-qPCR, PDX

## Abstract

**Background:**

We aim to characterize the heterogeneous circulating tumour cells (CTCs) in peripheral blood, independently of physical or immunological purification, by using patient-derived xenografts (PDXs) models. CTC studies from blood generally rely on enrichment or purification. Conversely, we devised a method for the inclusive study of human cells from blood of PDX models, without pre-selection or enrichment.

**Methods:**

A qRT-PCR assay was developed to detect human and cancer-related transcripts from CTCs in PDXs. We quantified the EPCAM and keratins CTC markers, in a PDX cohort of breast cancer. The murine beta actin gene was used for normalization. Spearman’s rho coefficients were calculated for correlation.

**Results:**

We demonstrated, for the first time, that we can quantify the content of CTCs and the expression of human CTC markers in PDX blood using human-specific qRT-PCR. Our method holds strong potential for the study of CTC heterogeneity and for the identification of novel CTC markers.

**Conclusions:**

The identification and the relative quantification of the diverse spectrum of CTCs in patients, irrespective of EPCAM or other currently used markers, will have a great impact on personalized medicine: unrestricted CTCs characterization will allow the early detection of metastases in cancer patients and the assessment of personalized therapies.

**Electronic supplementary material:**

The online version of this article (10.1186/s40364-018-0145-8) contains supplementary material, which is available to authorized users.

## Background

Metastases from primary tumours account for the great majority of cancer-related deaths [1]. This process is thought to involve a series of sequential steps, including the release of circulating tumour cells (CTCs) into blood stream [1, 2]. CTCs have been detected in the peripheral blood of patients with advanced cancers of different origin [3]. Recently, liquid biopsy focusing on the analysis of CTCs has received enormous attention because of its clinical implications [4–6]. The elucidation of CTC’s role might represent a turning point in understanding the evolution of tumours during treatment and progression [3]. Clinical applications of CTC detection include early cancer diagnosis, risk prediction for relapse or progression, monitoring the effects of systemic therapies, and stratification of patients [4]. CTC analysis might provide an alternative route for primary diagnosis of tumours, and for restaging/molecular analysis of metastatic lesions. At present, CTCs are used as biomarkers in > 270 clinical trials registered at ClinicalTrials.gov [5]. In on-going interventional studies, the clinical utility of CTC use for therapeutic decisions is being evaluated [7].

To date, however, only incomplete information is available on CTC molecular and biological heterogeneity, and significance. The fact that CTCs are extremely rare, even in patients with advanced metastatic cancers (estimated at one CTC/ billion normal blood cells), and that they may be poised on the verge of apoptosis, has made their analysis contingent upon technological constraints, posing a serious challenge to any analytical system [8–10]. Thus, although the potential CTC applications appear very promising, there is a critical need for increased detection sensitivity, requiring appropriate, high throughput, and reliable platforms for the isolation or rare tumour cells from blood.

It is thought that only a minor and variable fraction of CTCs represents viable metastatic precursors or tumour-initiating cells [11]. Aberrant activation of epithelial-mesenchymal transition (EMT) in adherent epithelial cells has been implicated in preclinical models as a prerequisite for invasive growth and metastatic spread [12–14]. EMT results in a reduced expression of epithelial markers, increased plasticity and capacity for migration and invasion, thus enabling detachment of tumour cells from a primary site and their passage into the circulation [12]. It also reduces their competence to initiate overt metastasis [15]. Therefore, the most accepted view is currently that tumour cells with a partial EMT have the highest plasticity and represent tumour cells with stem cell-like properties (i.e. metastatic potential) [4, 16, 17]. The detection of CTCs with such an intermediate phenotype seems to be now of the utmost importance, and thus the definition of a wider range of specific markers is required. In general, this plastic phenotype is characterized by partial down-regulation of epithelial markers, and partial up-regulation of mesenchymal markers [5]. Overall, thus far, there is still an incomplete knowledge on the heterogeneity of CTCs [18, 19]; further molecular characterization may give better insights, and help to distinguish clinically relevant CTCs [20]. Most of the current CTC isolation techniques rely on antibody-based capture. Epithelial markers expressed by tumours but not on blood cells have been frequently used to purify cancer cells [6, 21]. The CellSearch system (Menarini Silicon Biosystems), is highly standardized and is currently being evaluated for clinical applications, with CTCs defined as the subset of EpCAM (epithelial cell adhesion molecule)-captured cells, that are confirmed as positive for cytokeratins (KRT8, 18, and 19) and negative for CD45 [22]. However, this technique suffers from relatively low sensitivity: only a fraction of patients with metastatic cancer scores positive for CTCs, with a median yield of approximately one CTC per millilitre and typically low purity [23]. Thus, while progress has been made in the development of microfluidic devices, the discovery and validation of CTC markers is still in its infancy. Indeed, studies on CTCs have been hampered by reliance on a restricted pool of markers [3, 24], often leading to false-negative results [25]. To address these technical challenges, methods for the detection of CTCs from clinical samples should measure both the epithelial and the mesenchymal-like populations, as well as tumour cells with stem cell-like properties [16, 26]. In other words, there is a need for a broad-spectrum enrichment of CTCs based on cell surface epithelial and mesenchymal markers [12, 27], to avoid loss of CTCs with high phenotypic plasticity.

Here, we describe a novel approach that does not rely on the use of epithelial markers such as EPCAM for detection of CTCs in patient-derived xenografts (PDXs). PDXs are generated by transplanting patients’ tumour cells or tissues, obtained either by surgical resection or biopsy, into immunodeficient mice [28, 29]. The development of PDXs has significantly enhanced cancer research: these models have been established for different types of cancers, are biologically stable and reflect the patients’ tumours in terms of histology, gene profiles, inflammation, and therapeutic response. Thus, PDX models found general application in preclinical screening and evaluation of drugs, identification of therapeutic targets and biomarkers, biological studies, and personalized medicine strategies [29]. At present, CTCs have also been used to generate PDX models, with a great potential for studying tumorigenicity, and for the phenotypic and genetic characterization of CTCs themselves [29]. In this study, we took advantage of PDXs to study human CTCs in a murine genetic background, using human-specific RT-quantitative PCR (RT-qPCR) on RNA from total blood mononuclear cells. By using this highly specific and sensitive approach, without any CTC pre-isolation, we could identify and measure human CTC mRNAs irrespectively of the EPCAM content. In turn this procedure has the potential to identify membrane proteins, that could represent molecular handles for the immune-isolation of viable CTCs. Our approach could represent a major step towards the complete coverage of molecular and cellular heterogeneity in CTCs.

## Methods

### Patient-derived xenograft models establishment and blood samples collection

All PDXs used in the study were established upon approval of hospitals’ ethical committees, and an informed consent was signed for each patient. The major clinical characteristics of the PDX models used in this study are reported in Additional file 1: Table S1. Tumour samples were grafted in the interscapular region of 6- to 8-week-old female athymic nude mice (Athymic Nude-Foxn1nu, ENVIGO-Harlan Laboratories, Gannat, France). Growing tumours were serially transplanted onto recipient mice, underwent comparative examination to confirm preservation of their histological features and were live-frozen to immortalize the model. Blood was collected from mice with tumours reaching the ethical size (tumour volume > 1500 mm^3^) and eventually sacrificed in compliance with the indications provided in the protocol, as approved by the animal ethical committee and by the French Ministry of agriculture food and forestry. Briefly, at least 600–800 μl of whole blood were collected from mice by intra-cardiac sampling and under xylazine-ketamine anaesthesia. Blood samples were transferred in Eppendorf tubes containing 0.5 M EDTA, and erythrocytes were lysed at room temperature using the ACK Lysing Buffer (Lonza Walkersville, Inc., Allendale, NJ, USA). PBMC (Peripheral Blood Mononuclear Cells) were frozen in liquid nitrogen and stored at − 80 °C.

### RNA isolation and species-specific primers design

For gene expression studies, mouse or PDX PBMC, with or without human CTCs, were lysed by adding 500 μl of Trizol (Invitrogen, Carlsbad, CA, USA). RNA quantity and purity were assessed using a NanoDrop ND-1000 Spectrophotometer (Thermo Fischer Scientific, Wilmington, DE, USA). Total RNA (~ 1 μg) was reverse-transcribed in a final volume of 20 μl using random hexamers and SuperScript II (Life Technologies, Carlsbad, CA, USA). For each gene, human-specific primers were designed using Primer-BLAST at the National Center for Biotechnology Information and verified using the Genome Browser UCSC In-Silico PCR tool; primers with sequence homologies between *Homo sapiens* and *Mus musculus* were excluded. If possible, primers that amplified regions spanning two or more exons or the 5′- or 3’-UTR regions of the transcript were selected, to avoid amplification of genomic sequences and operator or laboratory contamination (Additional file 2: Table S2). To validate species-specificity, all oligonucleotides were tested in RT-PCR on RNA pools from human cell lines (ZR-75-1, MCF-7, MDA-MB-231 breast cancer, and MDA-MB-435 melanoma cells) and Sv/129 mouse blood. One μl of cDNA was amplified using DreamTaq DNA Polymerase (Life Technologies), according to the manufacturer’s instructions. Single nested PCR was performed with 1 μl of a 1:10 diluted pre-amplification reaction, by using DreamTaq DNA Polymerase (Life Technologies). PCR products were then analyzed on a 2% low melting point agarose gel.

### Quantitative PCR

Gene specific primers and conditions used in qPCR are listed in Additional file 2: Table S2. To ensure detection of very rare human transcripts, one μl of each cDNA was subjected to 14-cycles pre-amplification with DreamTaq DNA Polymerase, and human specific oligonucleotides: for both human reference and target transcripts, pre-amplification was performed with annealing at 58 °C for 5 min. qPCR was then performed using nested specific primers and Dual-Lock™ Taq DNA polymerase on a CFX96 Touch (Bio-Rad Laboratories, Inc., Hercules, CA, USA). qPCR was carried out in 10 μl volume using PowerUp SYBR Green Master Mix (Applied Biosystems™), and 1 μl of a 1:10 diluted pre-amplification reaction. Primers were added to a final concentration of 0.2 μM or 0.3 μM each, depending on the gene, as detailed in Additional file 2: Table S2. After the initial step (2 min at 50 °C for polymerase activation and 2 min of denaturation at 95 °C), 40 cycles were carried out by denaturation at 95 °C for 15 s, and annealing/extension as indicated in Additional file 2: Table S2. The melting curves were evaluated, to confirm the specificity of each reaction. Samples were analyzed in duplicate for mouse Actb reference (mActb), while for each human transcripts qPCR was repeated in four or five independent assays. RNAs from naïve mouse blood (negative control) and human cell lines (positive control) were included in each PCR assay.

The method based on the expectation–maximization (EM) algorithm, as implemented in the R ‘*nondetects’* package, was applied to estimate missing C_t_ values [30]. This function returns a qPCRset object with non-detects replaced by their imputed values. Non-detects in qPCR data represent data missing not at random, and the EM algorithm provides a method to obtain maximum likelihood estimates in the presence of missing data by iteratively calculating the conditional expectation. This requires estimating the distribution of gene expression given a non-detect; the process is repeated until convergence. Lastly, estimates of the missing data will be obtained and used to impute the non-detect values. PDX samples with at least one replicate for each human reference, having estimated C_t_ value larger than 40, were deemed as CTC-negative and excluded from further analyses. Estimated C_t_ values for target genes higher than 40 were thresholded to 40, and median C_t_ values used for each gene. The relative quantities of human reference genes normalized on the mouse Actb gene were calculated with the ΔC_t_ method using the R ‘*HTqPCR’* package. Conversely, for CTC relative measures, the quantities of human target genes were normalized on the quantities of each human reference gene. All statistical analyses, i.e. Spearman’s *rho* correlation coefficient calculation, were performed using SPSS (version 23) and *p* values were two-sided.

## Results

### Species-specific PCR for detection of human circulating tumour cells in PDX blood

**Fig. 1:**
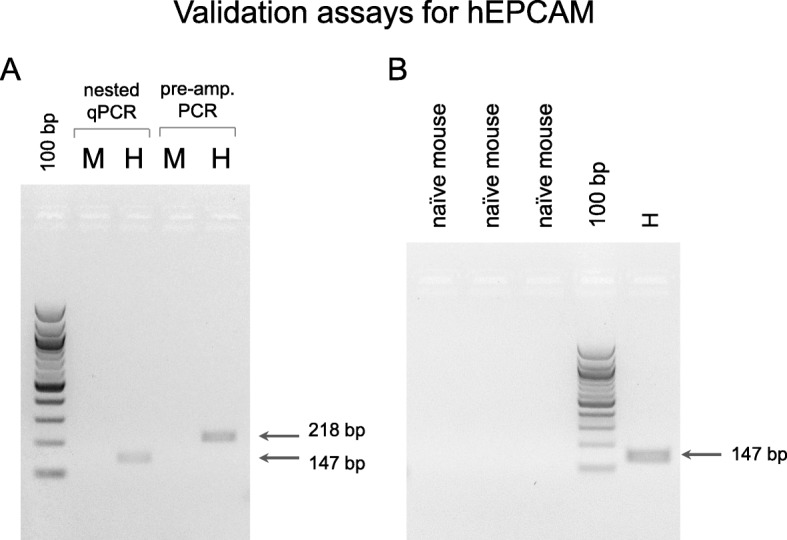
Representative results from RT- PCR assays for human EPCAM detection in PDX blood. **a** RT-PCR assays confirming PCR specificity on human gene were performed on pooled RNAs from human cell lines: ZR-75-1, MCF-7, MDA-MB-231 breast cancer, and MDA-MB-435 melanoma cells (H), and on RNA from Sv/129 mouse PBMC (M). Each primer set was tested at least in two independent assays. The expected size fragments are indicated. Molecular weight marker: 100 bp DNA Ladder (NE Biolabs). **b** The specificity of the nested-PCR assays was assessed on blood RNA from the naïve mouse used to generate the PDX models (naïve mouse) and on the pooled human cell lines RNA (H), used as negative and positive controls respectively. Each assay was performed at least twice. The expected size fragment is indicated. Molecular weight marker: 100 bp DNA Ladder (NE Biolabs)

Peripheral blood samples were collected from 28 PDXs and 3 naïve mice (negative controls). Following chemical erythrocytes depletion, RNA was extracted from the whole blood cell pellet, reverse-transcribed and tested for expression of the human EPCAM and KRT19 CTC markers. Human coding region-specific primers were also designed for two reference genes, OAZ1 and ACTB, to avoid detection of mouse homologues and amplification of human genomic DNA (Additional file 2: Table S2). The species-specificity of the protocol was assessed using naïve mouse blood samples and human cell lines as negative and positive controls, respectively. For primer optimization, cDNA from human cell lines and control mice was pre-amplified, and further subjected to single-gene nested PCR (representative assays are reported in Fig. 1). Once verified the species-specificity of the primers, the cDNA from the PDX cohort was tested for the expression of human genes.

### Human-specific quantitative RT-PCR on CTCs in blood of PDX mice

RNAs extracted from PDX blood samples were subjected to three independent reverse-transcription reactions and qPCR assays were performed on the pooled cDNAs. The expression levels of the murine reference gene Actb (mActb) and human reference genes OAZ1 and ACTB were measured in each sample. Human cDNAs were pre-amplified, and then subjected to nested-qPCR by using human-specific primers (Additional file 2: Table S2). No detection was obtained in control mice (no PDX) for both human reference genes (no control mice had C_t_ lower than 40), indicating that in our protocol the human specific quantitative PCR did not lead to false positives. The human references were amplified in five independent qPCRs for each sample (replicated). Undetermined C_t_ values were estimated by using the expectation–maximization (EM) algorithm, as previously described [30]. The blood from three PDX mice, HB-214-FOI(b), T180R(c), HBCx-28(a), was negative for the human reference genes, therefore had no detectable CTC and the samples were thus excluded from further studies.

### Expression of human CTC markers in blood from PDX mice

**Fig. 2:**
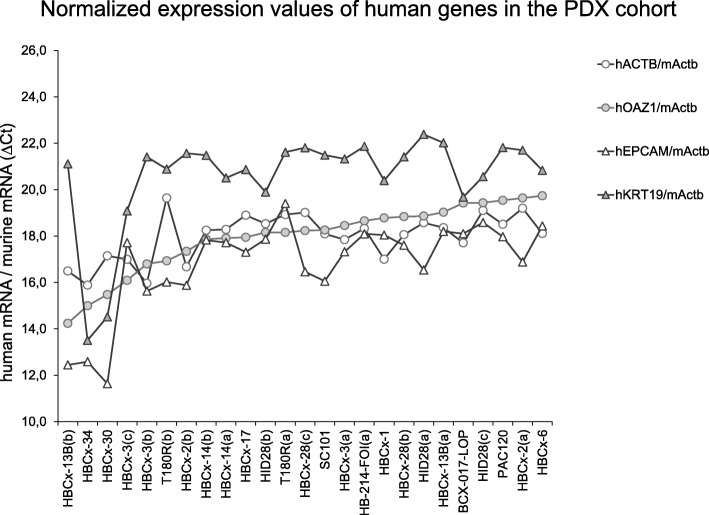
Normalized expression levels of human genes. The expression levels of human ACTB and OAZ1 reference genes and EPCAM and KRT19 epithelial markers were normalized on murine reference gene Actb, for each PDX sample. Data were expressed as ΔCt values and graphed, sorted on human OAZ1 content. Samples were analyzed in two independent assays for mActb; for human transcripts, qPCR was repeated in four (KRT19) or five (ACTB, OAZ1, EPCAM) independent assays for each gene. In each run, each RNA was measured in duplicate

**Fig. 3:**
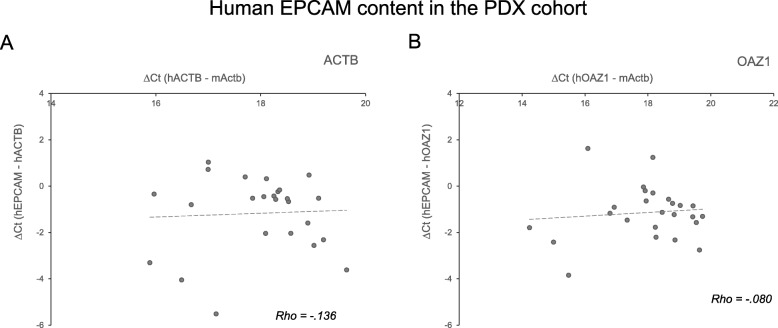
Correlation of EPCAM expression and human RNA content in PDX cohort. The correlation between EPCAM expression relative to human references ACTB (in **a**) or OAZ1 (in **b**) and the human reference content relative to murine Actb was graphed. Each PDX blood sample gene expression levels were graphed as a scatterplot. Human reference gene Ct values were normalized on the murine Actb gene. Human EPCAM Ct values were normalized on human reference gene. The linear regression fit lines and Spearman’s rho correlation coefficients are reported

**Fig. 4:**
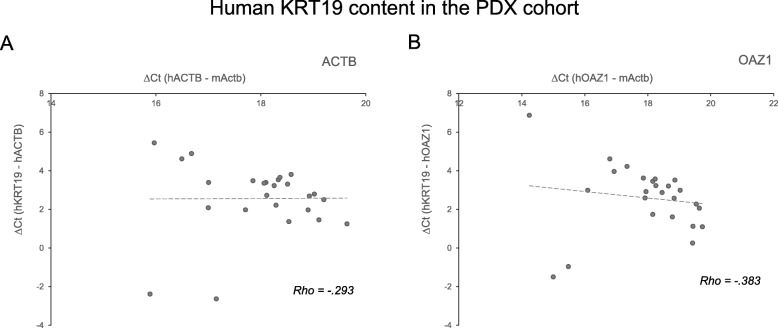
Correlation of KRT19 expression and human RNA content in PDX cohort. The correlation between KRT19 expression relative to human references ACTB (in **a**) or OAZ1 (in **b**) and the human reference content relative to murine Actb was graphed. Each PDX blood sample gene expression levels were graphed as a scatterplot. Human reference gene Ct values were normalized on the murine Actb gene. Human KRT19 Ct values were normalized on human reference gene. The linear regression fit lines and Spearman’s rho correlation coefficients are reported

The expression levels of human EPCAM, KRT19, ACTB and OAZ1 in the blood of PDX models were measured using qPCR (Additional file 3: Table S3 and Additional file 4: Table S4). The normalized expression values for human genes were calculated relative to murine Actb and shown in Fig. 2, sorted by the level of the human OAZ1 reference gene (as approx. indicator of CTC content). As expected for two ‘housekeeping’ genes, the RNA levels of the two human reference genes (ACTB and OAZ1) were significantly correlated (Spearman’s *rho* = 0.462, *p* < 0.05). Ideally ACTB, OAZ1, or any other human ʻhousekeepingʼ gene, would act as proxies for the assessment of human CTC content in the PDX blood. We then studied the expression of EPCAM and KRT19 epithelial markers within the human CTC component. The normalized expression values for human target genes were calculated relative to each human reference gene, using the ΔC_t_ method, obviously with no difference whether the human genes were previously normalized on murine Actb. The expected plots for the different hypotheses that EPCAM levels were not (A), positively (B) or negatively (C) correlated with the total human RNA content are shown in Additional file 5: Figure S1, and describe the predictions of EPCAM profiles relative to human OAZ1. Because the plots report ΔC_t_ values, the PDX samples with higher CTC content (more RNA from OAZ1 housekeeping gene) are towards the left, and those with higher EPCAM in CTC are towards the bottom. The null hypothesis was that EPCAM, if present, was expressed by the CTCs at constant cellular levels (RNA molecules per CTC cell) in different PDXs; thus as human RNA content increases, EPCAM levels should be correspondingly higher (resulting in relatively constant ΔC_t_(hEPCAM - hOAZ1) values); this would correspond to the panel A in Additional file 5: Figure S1 (flat line, no correlation). In Fig. 3 we report the real data for measured EPCAM levels, relative to two human reference RNAs. In the PDX cohort, the EPCAM levels showed to be relatively constant, as the human content increased, indicative of a pattern similar to A in Additional file 5: Figure S1 (stable reservoir of EPCAM+ cells). The same protocol was applied to KRT19, the most common alternative marker for CTCs detection. As observed for EPCAM, also KRT19 was expressed at similar content in PDXs with diverse amounts of human reference RNAs (Fig. 4). No significant correlation was measured between the two epithelial markers (Spearman’s rho = 0.219, relative to ACTB, and rho = 0.152, relative to OAZ1; both *p* > 0.05) [31].

## Discussion

Detection of circulating tumour cells in the blood of patients with cancer shows great potential as a ‘liquid biopsy’, and multiple technologies have been developed for CTC capture [4]. The limitations in current CTC detection are mainly due to reduced specificity and sensitivity and should be carefully considered when interpreting the literature [3]. The quest for technical developments has slowed down the introduction of CTCs into clinical diagnostics. However, new important insights into the biology of CTCs have been reported [32], and tools for combined enrichment, detection, and characterization of CTCs are emerging.

Although the prognostic value of CTC has been demonstrated [33–35], metastatic patients frequently present little or undetectable CTCs when using epithelial markers [12]. Otherwise, higher numbers of CTCs could be detected using alternative methods, suggesting that a mixture of EPCAM+ and EPCAM- tumour cells circulate in the blood [12, 36]. Therefore EpCAM-based CTC isolation might suffer of limitations in the clinical settings since its sensitivity is significantly dependent on EpCAM expression [37]. Also, non-epithelial CTCs might exert a more relevant function in tumorigenesis: indeed, mouse models and in vitro experiments evidenced that EMT, together with stem-like properties in circulating cancer cells, is associated with increased cellular migration and resistance to therapy [16, 38]. Thus, the pre-selection for cells that have retained significant expression of epithelial markers represents an intrinsic challenge in studying CTCs [27]. To overcome these limitations the isolation of CTCs based on size has been described [8], reportedly leading to higher CTC yields. A larger set of tumour-specific markers would be then useful in establishing the identity of the heterogeneous CTCs enriched by filtration strategies, and to rescue those that might escape.

To develop a method for the identification of undifferentiated (or EPCAM-) CTCs, we used human specie-specific qRT-PCR and blood from PDXs. PDX models represent an ideal system for our study, allowing to exclusively profile the human transcriptome in the CTCs, excluding a priori the possible contamination of human mononuclear cells. Species-specific primers were designed and validated in human cell lines and naïve mouse blood. Nested quantitative human-specific qRT-PCR was established as a highly specific and sensitive method to detect and measure the expression of human CTC genes, with no detection of murine genes. Human RNAs for reference genes and epithelial markers were quantified in the blood of patient-derived xenografts from breast cancer. The null hypothesis in our study was that EPCAM, when present, would be expressed in CTCs at levels correlated to those of the human housekeeping genes. These results were obtained by using two different reference genes, OAZ1 and ACTB (found to be correlated between them, as expected for housekeeping genes), and also mirrored by another epithelial marker, KRT19. Our findings confirmed that we could reliably measure mRNA from rare CTCs, in the peripheral blood, with the highest degree of sensitivity and specificity. Therefore our assay is able to determine the presence of occult circulating tumour cells in the peripheral blood and has the potential to identify novel CTC markers using PDX mice. For the accurate measure of human CTC content in PDX blood, genomic DNA would be used. Tumour DNA content is more stable than RNA content, thus in principle this measure could result in absolute CTC counts. We decided to focus at first on human mRNA to avoid any environmental contamination, which could greatly affect the results of this extremely sensitive protocol.

The follow-up of this pilot study involves: *i)* identification of novel CTC markers from PDX cohorts; *ii)* test of these novel CTC markers in blood from human cancer patients. Genes detected in the EPCAM-negative subset of samples would lead to isolate CTC subpopulations with intermediate or not well defined phenotype. Eventually a novel set of genes for CTCs capture would be identified, allowing for capture of viable heterogeneous CTCs in murine human cancer xenograft models. Transcripts with no, or very low, expression in PBMC from healthy donors could be used for CTC monitoring in blood of tumour patients for clinical and research applications. At last, human genes expressed in blood of PDX models, originating from circulating tumour cells, will need to be validated in human blood specimens. The application of our approach was originally addressed to the detection and capture of human circulating tumour cells deriving from solid tumours, first in PDX models and then in patients. However, it can be applied in PDX models also for human hematologic malignancies. Recently, a model of human AML has been developed, which efficiently recapitulated the pathological features, including myeloid sarcoma, spleen enlargement, and infiltration of leukemic cells from bone marrow [39]. Since our method is designed to discriminate the transcripts from murine and human species in murine blood samples, circulating leukemic stem cells, which are believed to influence engraftment potential in recipient mice and are responsible for disease resistance or relapse in patients, could be easily examined.

## Conclusions

It is in our plans to extend the number of human CTC markers in the assay, the cohort of PDX mice, to incorporate CTC counting by genomic DNA and to perform in parallel single-cell RNA sequencing. Using multiple targets for CTC capture and identification would increase the comprehensiveness of CTC detection. Our work shows the presence of heterogeneous molecular phenotypes in CTCs from a PDX breast cancer cohort and provides the basis for the detection of the diverse spectrum of tumour cells that circulate in blood. Ultimately, alternative methods for detection of CTCs from human samples would be implemented. Extended molecular characterization of CTCs may provide further insight into the biology of these cells and their link to metastasis. Furthermore, molecular profiles of CTCs could lead to novel non-invasive diagnostic tools, and allow real-time monitoring of treatment responses. In this context, we consider our protocol as a proof of concept, with a great potential in opening up a new way towards the identification of novel markers for CTC capture.

## Additional files

Additional file 1: **Table S1.** Description of human tumours grafted to generate PDX models, and their main clinical features. (XLSX 12 kb)

Additional file 2: **Table S2.** Description of oligonucleotides used for multiplex- and nested-gene-specific RT-PCR assays. (XLSX 11 kb)

Additional file 3: **Table S3.** Quantitative PCR data of all genes analyzed for each sample in the PDX cohort. (XLSX 58 kb)

Additional file 4: **Table S4.** Quantitative PCR data, including computed values, for each sample analyzed in the PDX cohort. (XLSX 13 kb)

Additional file 5: **Figure S1.** Simulation and real data of human EPCAM RNA detection in CTC from PDX blood. The expression values of EPCAM in CTCs (relative to human reference OAZ1), as calculated for 3 different trends are plotted: A, no variation of relative EPCAM levels among samples; B, ~ 10% EPCAM levels increasing faster than human RNA content; C, ~ 10% decreasing EPCAM faster than human RNA content. The PDX samples with higher CTC content (more RNA from OAZ1 reference gene) are towards the left and those with higher EPCAM in CTC are towards the bottom. The measured EPCAM relative to human reference OAZ1 and the human OAZ1 CTC levels, normalized on murine Actb, are shown in panel D (from 5 independent qPCR experiments). The linear regression fit lines are reported. (PDF 30 kb)

## Abbreviations

ACTB: Actin beta [*Homo sapiens*]
CTC: Circulating tumour cells
EPCAM: Epithelial cell adhesion molecule [*Homo sapiens*]
KRT19: Keratin 19 [*Homo sapiens*]
mActb: Actin beta [*Mus musculus*]
OAZ1: Ornithine decarboxylase antizyme 1 [*Homo sapiens*]
PDX: Patient-derived xenografts

## References

[1] Nguyen DX, Bos PD, Massagué J (2009). Metastasis: from dissemination to organ-specific colonization. Nat Rev Cancer.

[2] Aceto N, Bardia A, Miyamoto DT, Donaldson MC, Wittner BS, Spencer JA (2014). Circulating tumor cell clusters are oligoclonal precursors of breast cancer metastasis. Cell.

[3] Yu M, Stott S, Toner M, Maheswaran S, Haber DA (2011). Circulating tumor cells: approaches to isolation and characterization. J Cell Biol.

[4] Alix-Panabières C, Pantel K (2016). Clinical applications of circulating tumor cells and circulating tumor DNA as liquid biopsy. Cancer Discov.

[5] Alix-Panabières C, Pantel K (2014). Challenges in circulating tumour cell research. Nat Rev Cancer.

[6] Pantel K, Alix-Panabières C (2010). Circulating tumour cells in cancer patients: challenges and perspectives. Trends Mol Med.

[7] Bidard F-C, Fehm T, Ignatiadis M, Smerage JB, Alix-Panabières C, Janni W (2013). Clinical application of circulating tumor cells in breast cancer: overview of the current interventional trials. Cancer Metastasis Rev.

[8] Nagrath S, Sequist LV, Maheswaran S, Bell DW, Irimia D, Ulkus L (2007). Isolation of rare circulating tumour cells in cancer patients by microchip technology. Nature.

[9] Ozkumur E, Shah AM, Ciciliano JC, Emmink BL, Miyamoto DT, Brachtel E (2013). Inertial focusing for tumor antigen-dependent and -independent sorting of rare circulating tumor cells. Sci Transl Med.

[10] Stott SL, Hsu C-H, Tsukrov DI, Yu M, Miyamoto DT, Waltman BA (2010). Isolation of circulating tumor cells using a microvortex-generating herringbone-chip. Proc Natl Acad Sci U S A.

[11] Hodgkinson CL, Morrow CJ, Li Y, Metcalf RL, Rothwell DG, Trapani F (2014). Tumorigenicity and genetic profiling of circulating tumor cells in small-cell lung cancer. Nat Med.

[12] Yu M, Bardia A, Wittner BS, Stott SL, Smas ME, Ting DT (2013). Circulating breast tumor cells exhibit dynamic changes in epithelial and mesenchymal composition. Science.

[13] Pecot CV, Bischoff FZ, Mayer JA, Wong KL, Pham T, Bottsford-Miller J (2011). A novel platform for detection of CK+ and CK- CTCs. Cancer Discov.

[14] Thiery JP (2002). Epithelial-mesenchymal transitions in tumour progression. Nat Rev Cancer.

[15] Kang Y, Pantel K (2013). Tumor cell dissemination: emerging biological insights from animal models and cancer patients. Cancer Cell.

[16] Mani SA, Guo W, Liao M-J, Eaton EN, Ayyanan A, Zhou AY (2008). The epithelial-mesenchymal transition generates cells with properties of stem cells. Cell.

[17] Tam WL, Weinberg RA (2013). The epigenetics of epithelial-mesenchymal plasticity in cancer. Nat Med.

[18] Wan L, Pantel K, Kang Y (2013). Tumor metastasis: moving new biological insights into the clinic. Nat Med.

[19] Lohr JG, Adalsteinsson VA, Cibulskis K, Choudhury AD, Rosenberg M, Cruz-Gordillo P (2014). Whole-exome sequencing of circulating tumor cells provides a window into metastatic prostate cancer. Nat Biotechnol.

[20] Wicha MS, Hayes DF (2011). Circulating tumor cells: not all detected cells are bad and not all bad cells are detected. J Clin Oncol.

[21] Pantel K, Alix-Panabières C (2013). Real-time liquid biopsy in cancer patients: fact or fiction?. Cancer Res.

[22] Riethdorf S, Fritsche H, Müller V, Rau T, Schindlbeck C, Rack B (2007). Detection of circulating tumor cells in peripheral blood of patients with metastatic breast cancer: a validation study of the CellSearch system. Clin Cancer Res.

[23] Attard G, Swennenhuis JF, Olmos D, Reid AHM, Vickers E, A’Hern R (2009). Characterization of ERG, AR and PTEN gene status in circulating tumor cells from patients with castration-resistant prostate cancer. Cancer Res.

[24] Pantel K, Brakenhoff RH, Brandt B (2008). Detection, clinical relevance and specific biological properties of disseminating tumour cells. Nat Rev Cancer.

[25] Bednarz-Knoll N, Alix-Panabières C, Pantel K (2011). Clinical relevance and biology of circulating tumor cells. Breast Cancer Res.

[26] Aktas B, Tewes M, Fehm T, Hauch S, Kimmig R, Kasimir-Bauer S (2009). Stem cell and epithelial-mesenchymal transition markers are frequently overexpressed in circulating tumor cells of metastatic breast cancer patients. Breast Cancer Res.

[27] Sieuwerts AM, Kraan J, Bolt J, van der Spoel P, Elstrodt F, Schutte M (2009). Anti-epithelial cell adhesion molecule antibodies and the detection of circulating normal-like breast tumor cells. J Natl Cancer Inst.

[28] Hidalgo M, Amant F, Biankin AV, Budinská E, Byrne AT, Caldas C (2014). Patient-derived xenograft models: an emerging platform for translational cancer research. Cancer Discov.

[29] Aparicio S, Hidalgo M, Kung AL (2015). Examining the utility of patient-derived xenograft mouse models. Nat Rev Cancer.

[30] McCall MN, McMurray HR, Land H, Almudevar A (2014). On non-detects in qPCR data. Bioinformatics.

[31] Mikolajczyk SD, Millar LS, Tsinberg P, Coutts SM, Zomorrodi M, Pham T (2011). Detection of EpCAM-negative and cytokeratin-negative circulating tumor cells in peripheral blood. J Oncol.

[32] Pantel K, Speicher MR (2016). The biology of circulating tumor cells. Oncogene.

[33] Cohen SJ, Punt CJA, Iannotti N, Saidman BH, Sabbath KD, Gabrail NY (2009). Prognostic significance of circulating tumor cells in patients with metastatic colorectal cancer. Ann Oncol.

[34] Cristofanilli M, Budd GT, Ellis MJ, Stopeck A, Matera J, Miller MC (2004). Circulating tumor cells, disease progression, and survival in metastatic breast cancer. N Engl J Med.

[35] de Bono JS, Scher HI, Montgomery RB, Parker C, Miller MC, Tissing H (2008). Circulating tumor cells predict survival benefit from treatment in metastatic castration-resistant prostate cancer. Clin Cancer Res.

[36] Thurm H, Ebel S, Kentenich C, Hemsen A, Riethdorf S, Coith C (2003). Rare expression of epithelial cell adhesion molecule on residual micrometastatic breast cancer cells after adjuvant chemotherapy. Clin Cancer Res.

[37] Gorges TM, Tinhofer I, Drosch M, Röse L, Zollner TM, Krahn T (2012). Circulating tumour cells escape from EpCAM-based detection due to epithelial-to-mesenchymal transition. BMC Cancer.

[38] Polyak K, Weinberg RA (2009). Transitions between epithelial and mesenchymal states: acquisition of malignant and stem cell traits. Nat Rev Cancer.

[39] Her Z, Yong KSM, Paramasivam K, Tan WWS, Chan XY, Tan SY (2017). An improved pre-clinical patient-derived liquid xenograft mouse model for acute myeloid leukemia. J Hematol Oncol.

