# Supplementary material for: Heterogeneous expression of EPCAM in human circulating tumour cells from patient-derived xenografts
Source: Biomark Res. 2018 Oct 30;6:31. doi: 10.1186/s40364-018-0145-8 (PMC6208170; doi:10.1186/s40364-018-0145-8)
Supplement: Supplementary file 5 — Figure S1. Simulation and real data of human EPCAM RNA detection in CTC from PDX blood. The expression values of EPCAM in CTCs (relative to human reference OAZ1), as calculated for 3 different trends are plotted: A, no variation of relative EPCAM levels among samples; B, ~ 10% EPCAM levels increasing faster than human RNA content; C, ~ 10% decreasing EPCAM faster than human RNA content. The PDX samples with higher CTC content (more RNA from OAZ1 reference gene) are towards the left and those with higher EPCAM in CTC are towards the bottom. The measured EPCAM relative to human reference OAZ1 and the human OAZ1 CTC levels, normalized on murine Actb, are shown in panel D (from 5 independent qPCR experiments). The linear regression fit lines are reported. (PDF 30 kb) [file 40364_2018_145_MOESM5_ESM.pdf]

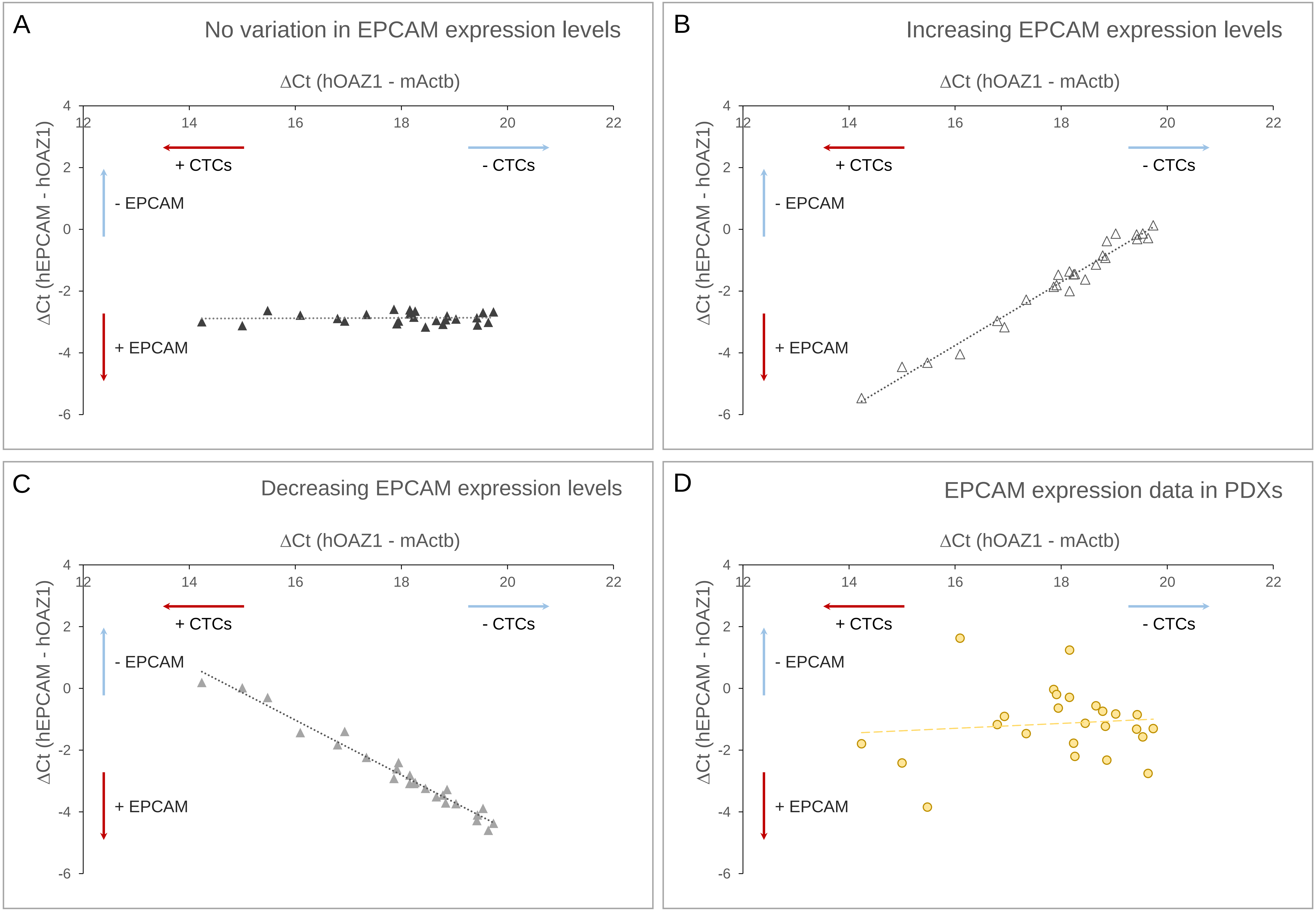
A
No variation in EPCAM expression levels
B
Increasing EPCAM expression levels
C
Decreasing EPCAM expression levels
D
EPCAM expression data in PDXs
ΔCt (hOAZ1 - mActb)
ΔCt (hEPCAM - hOAZ1)
+ CTCs
- CTCs
- EPCAM
+ EPCAM
12
14
16
18
20
22
4
2
0
-2
-4
-6
